# Acute Mastoiditis in a Newborn with Aural Atresia

**DOI:** 10.1155/2012/459293

**Published:** 2012-08-07

**Authors:** K. Parpounas, K. Bouka, J. Athanasopoulos, M. Lamprou, G. Helmis, I. M. Vlastos

**Affiliations:** ^1^ENT Clinic, 28 Voreiou Ipeirou, Kolonos, Athens 10444, Greece; ^2^Neonate Intensive Care Unit (A'NICU), Aghia Sophia Children's Hospital, Athens 11527, Greece

## Abstract

Acute mastoiditis in the newborn is a very rare disease. Herein we report a case of a 28-day-old child with right aural atresia and ipsilateral mastoiditis requiring mastoidectomy. To our knowledge, this is the youngest case reported in the literature. Issues on diagnosis and management of mastoiditis in cases of aural atresia are further discussed. Based on our experience and on previous reported cases we conclude that mastoiditis is difficult to diagnose in a child with aural atresia. Moreover, mastoidectomy may be necessary, although identification of the facial nerve and consequent treatment of the atresia are usually too difficult to perform simultaneously.

## 1. Introduction

Congenital aural atresia occurs approximately one in every 20 000 live births. Unilateral atresia is four times more common than bilateral atresia. Abnormalities of the middle and inner ear are associated with atresia in 20–30% of cases [1, 2].

Diagnosis of otitis media in children with congenital atresia is difficult to detect especially when the symptoms mimic very common infections of the upper respiratory system since no otoscopy is possible. In these cases, otitis complication, such as acute mastoiditis, usually reveals the real ear problem.

Although acute mastoiditis remains a relatively common clinical problem, it is extremely rare in the newborn. What is more, aural atresia represents a surgically challenging problem, both in technical terms and in relation to the appropriate treatment option, especially in cases of complications. This paper details the rare case of a four-week-old child with acute coalescent mastoiditis in an atretic ear. 

## 2. Case Report

**Figure 1:**
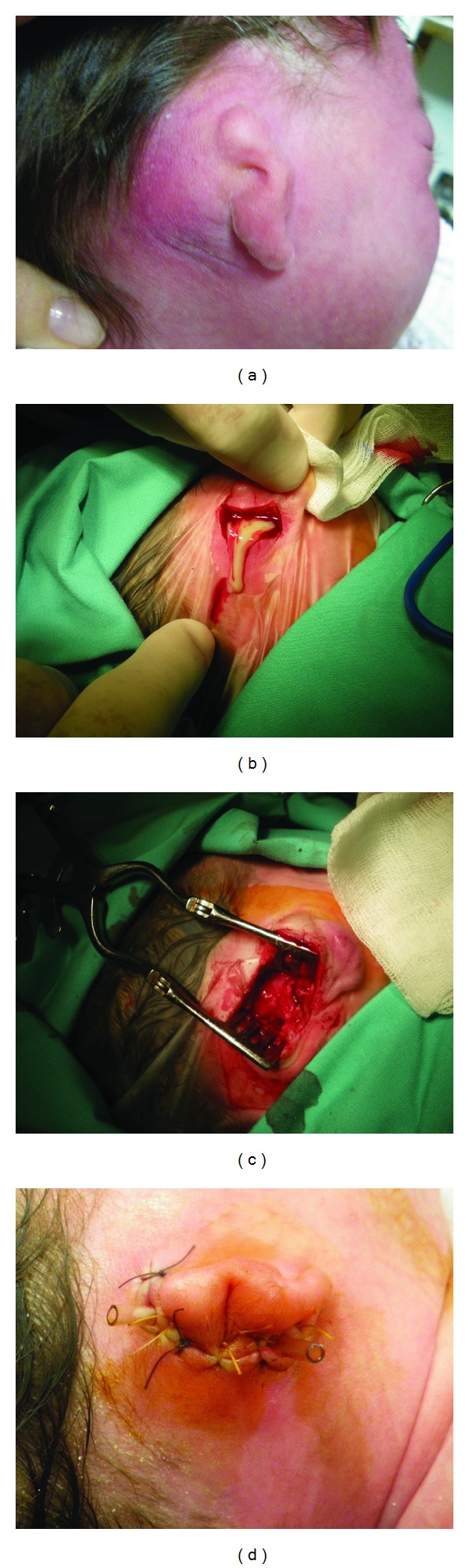
Pre- (a), intra- (b) and (c), and immediate postoperative (d) view of the atretic ear with the mastoiditis.

A 28-day-old female newborn with right congenital aural atresia was referred to our emergency department with postauricular erythema and edema, accompanied by irritability, anorexia, and insomnia, without fever, since two days. On initial examination, the newborn had a small, deformed auricular tag on the right side with no external auditory meatus. She presented with edema, erythema, and tenderness over the right mastoid expanding to the zygomatic bone area (Figure 1(a)). Her left ear as well as her rest physical examination were unremarkable. Her weight was 3780 gr.

**Figure 2:**
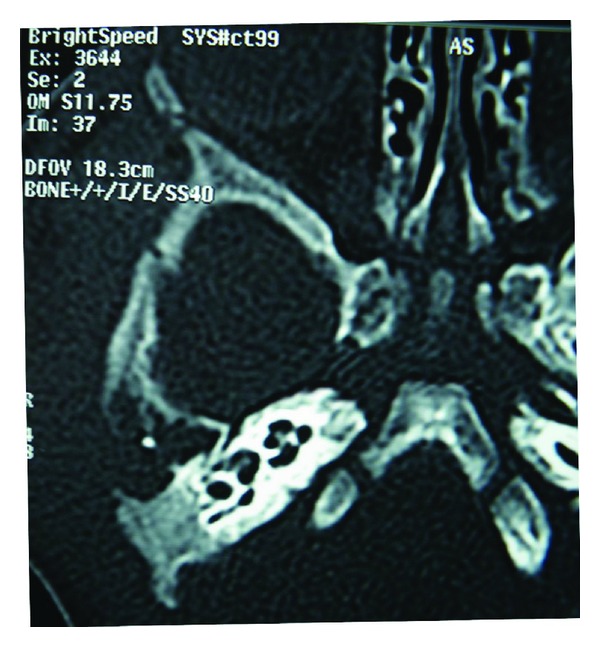
CT scan showing bony lytic lesions.

The child had increased inflammation markers (white blood cells: 19.900/*μ*L and CRP: 111 mg/L). MRI and high-resolution CT scans (Figure 2) of the head and temporal bone showed dysplasia of right petrosal bone, bony type atresia of the external auditory meatus with well-developed tympanic cavity. A mass of soft tissue density causing osteolytic lesions was extended from the parotid area to the temporal area, the subtemporal fossa, and the pterygoid muscle area in the right.

The newborn was started immediately on meropenem and vancomycin i.v. Two days later, her condition remained unchanced, and a mastoidectomy was performed. Right temporomantidular joint, zygomatic process of the temporal bone, and the mastoid tip were palpated and used as landmarks for a curvilinear incision over the mastoid bone. Abnormal bone was drilled or curetted away. Mastoid cells filled with granulation tissue were exenterated and about 2 mL of pus were collected for culture (Figures 1(b) and 1(c)). Pus and blood cultures were negative. Pathological examination of the bone and granulation tissue removed showed evidence of osteomyelitis. 

The antibiotic scheme continued for a total of four weeks. Her postoperative course was unremarkable, with progressive resolution of signs of inflammation on mastoid area. The baby girl remained afebrile and her alimentation as well as the inflammation markers improved gradually. She left the hospital in a very good condition upon completion of her treatment. The baby has no problem two months after her operation, and her weight is 5.100 gr.

## 3. Discussion

The presence of inflammation in the middle ear of atretic ears has been well documented since 1912 by Krampitz [3] who was the first to report a case of purulent middle ear disease associated with microtia [2]. Since upper respiratory infections are expected to ascend to the middle ear in a manner similar to that of patients with normal ears, a large number of ear infections in congenital atresia go undetected [1]. Thus the incidental finding of infection in surgically explored atretic ears should not be considered surprising. However the clinical presentation of coalescent mastoiditis in these cases is extremely rare [2, 4–7]. The youngest patient reported by the aforementioned studies is a 6-week-old child who was treated with surgical removal of the affected bone. 

In our case, an even younger child required surgical intervention. Despite current reports [8, 9] indicating that a great number of mastoidectomies can be avoided with proper antibiotic treatment, adverse events and no improvement are generally accepted indications for surgical intervention [9]. Moreover, due to the underdeveloped mastoid cavity of the newborn, CT scans can show a relatively large bone destruction that should be differentiated by entities such as sarcoma or Langerhans histiocytosis. 

Surgical treatment is challenging because of the anatomy, the presence of inflammation, and the very young age. Thus, identification of the facial nerve course in the inflamed and underdeveloped temporal bone may not be possible as in our case or in previous cases [1]. 

In conclusion, whether atresia is related to an increased incidence of ear infection is a subject of controversy and speculation. There are relatively large atresia series showing both increased [4] and normal [5] rates of infection in atretic ears. Nevertheless the evolution of infection into coalescent mastoiditis is a rare incidence. However, clinicians should take into consideration that, although rare, mastoiditis in congenital atresia cases is possible and can be presented even in a one-month-old child. Moreover, surgical intervention may be inevitable even though it has several difficulties and atresia correction is usually not possible simultaneously.
